# Adrenomedullin: a vasodilator to treat sepsis?

**DOI:** 10.1186/cc13924

**Published:** 2014-06-16

**Authors:** Jérôme Pugin

**Affiliations:** 1 Intensive Care Division, University Hospitals of Geneva, CH-1211 Geneva 14, Switzerland

## Abstract

Adrenomedullin is a vasodilatory polypeptide with pleiotropic effects secreted by various organs. Adrenomedullin is produced first as a prepropeptide, and then cleaved into mature adrenomedullin and mid-regional proadrenomedullin. Whereas levels of the latter have been shown to correlate with severity of sepsis and carry prognostic value, adrenomedullin plays a role in vascular tone homeostasis. In the previous issue of *Critical Care*, the infusion of exogenous adrenomedullin is suggested to protect against increased lung endothelial permeability and end-organ dysfunction in a model of pneumococcal pneumonia in mechanically ventilated mice, possibly by stabilizing vascular endothelia. Since adrenomedullin is a strong vasodilatory molecule, further studies are needed to evaluate its potential as a future treatment of sepsis.

## 

In the previous issue of *Critical Care*, Müller-Redetzky and colleagues propose that adrenomedullin may be promising to treat sepsis [1]. Adrenomedullin is a 52 amino acid polypeptide showing sequence homology with other calcitonin-related peptides, discovered 20 years ago as a secretion product of a pheochromocytoma. The adrenomedullin gene encodes for a 185 amino acid prepropeptide, post-transcriptionally cleaved to produce the bioactive, amidated adrenomedullin.

Adrenomedullin is found in plasma in health and at elevated concentrations during various pathologic conditions, including cardiovascular and renal disorders, sepsis, cancer, and diabetes [2]. This polypeptide has a potent vasodilating effect and is believed to play a physiological role in arterial pressure homeostasis. Adrenomedullin exerts its effects via its ligation to the G-coupled seven-transmembrane calcitonin receptor-like receptor (CALCRL or CLR) in association with RAMP2 or RAMP3 to form the adrenomedullin receptors 1 and 2, respectively. Numerous pleiotropic effects of adrenomedullin have been described: in tumor growth, neovascularization, endothelium protection, bronchodilation, fertility, and immunity [3].

Interestingly, the plasma concentration of a byproduct of the preproadrenomedullin cleavage, the mid-regional proadrenomedullin, has been used as a prognostic marker, alone or in risk stratification with other propeptides such as procalcitonin, in children and in adult patients with sepsis and severe pneumonia [4]. This biomarker is now commercially available.

Müller-Redetzky and colleagues propose that adrenomedullin treatment is beneficial in a mouse model of pneumococcal pneumonia and ventilator-induced lung injury [1]. In a previous report, the same group had already shown that adrenomedullin treatment attenuated ventilator-induced lung injury in mice [5]. As has already been shown in various rodent models, the addition of mechanical ventilation to pneumonia worsened the outcome, in particular by inducing severe lung injury, sepsis, and end-organ dysfunction [6,7]. In the study by Müller-Redetzky and colleagues, the infusion of adrenomedullin was associated with decreased alveolar barrier permeability, and protective effects on end organs such as the liver and gut, suggested by lower transaminase plasma levels and less cell death on histology [1]. Adrenomedullin treatment did not ameliorate the kidney function, however, and nor did it influence lung and blood bacterial burden or lung cytokine levels. Adrenomedullin infusion did not significantly impact on macrohemodynamics in this model. The authors postulate that most of the beneficial effects observed with adrenomedullin treatment could be due to a stabilization of endothelial integrity.

Preserving or restoring endothelial function in sepsis and lung injury has been a holy grail for a long period of time. Whereas the restoration of macrohemodynamics is usually achieved with fluid loading, vasopressors, and sometimes cardiotonic agents in patients with sepsis, microcirculation remains severely impaired, preventing adequate supply of oxygen and nutrients to tissues. The implication of endothelial cells is central to this syndrome. The cells control vascular tone in a nitric oxide-dependent manner - and therefore arterial pressure - and also control the permeability of capillaries. Endothelial cells are activated during the initial phase of sepsis, plugging capillaries with circulating leukocytes. The endothelium also becomes leaky due to disruption of intercellular junctions and endothelial cell death. Microcirculation improves with microbiological source control and resolution of the sepsis syndrome, the negativation of the fluid balance being a positive sign of sepsis resolution.

Adrenomedullin treatment offers hope for restoring endothelial stability because it may prevent undesired decompartmentalization of the inflammatory reaction present in infected organs, and in the lung in particular. One should, however, keep in mind that adrenomedullin is a strong vasodilatory molecule, which will certainly not be easy to use in shocked patients. In addition, blocking adrenomedullin has also been shown to have a positive impact in mice with cecal ligature and puncture, improving catecholamine responsiveness, blunting the shock-related impairment of energy metabolism, reducing nitrosative stress, and attenuating systemic inflammatory response [8]. Caution is therefore mandatory with the development of adrenomedullin as a drug, as with any new treatment for sepsis.

## Competing interests

The author declares that he has no competing interests.
